# Solving the Min-Max Clustered Traveling Salesmen Problem Based on Genetic Algorithm

**DOI:** 10.3390/biomimetics8020238

**Published:** 2023-06-06

**Authors:** Xiaoguang Bao, Guojun Wang, Lei Xu, Zhaocai Wang

**Affiliations:** College of Information Technology, Shanghai Ocean University, Shanghai 201306, China; xgbao@shou.edu.cn (X.B.); vienna_wa@163.com (G.W.); hy1502102@163.com (L.X.)

**Keywords:** traveling salesman problem, multiple traveling salesmen problem, clustered traveling salesman problem, min-max, genetic algorithm

## Abstract

The min-max clustered traveling salesmen problem (MMCTSP) is a generalized variant of the classical traveling salesman problem (TSP). In this problem, the vertices of the graph are partitioned into a given number of clusters and we are asked to find a collection of tours to visit all the vertices with the constraint that the vertices of each cluster are visited consecutively. The objective of the problem is to minimize the weight of the maximum weight tour. For this problem, a two-stage solution method based on a genetic algorithm is designed according to the problem characteristics. The first stage is to determine the visiting order of the vertices within each cluster, by abstracting a TSP from the corresponding cluster and applying a genetic algorithm to solve it. The second stage is to determine the assignment of clusters to salesmen and the visiting order of the assigned clusters. In this stage, by representing each cluster as a node and using the result of the first stage and the ideas of greed and random, we define the distances between each two nodes and construct a multiple traveling salesmen problem (MTSP), and then apply a grouping-based genetic algorithm to solve it. Computational experiments indicate that the proposed algorithm can obtain better solution results for various scale instances and shows good solution performance.

## 1. Introduction

The traveling salesman problem (TSP) is a classical combinatorial optimization problem in computer science and operations research. Given some cities to be served and the distance between each two cities, the problem requires computing a tour that can pass through all cities once and only once and has the shortest total travel distance. Since the TSP was introduced, it and its related variants have received extensive attention from many researchers. The clustered traveling salesman problem (CTSP) and multiple traveling salesmen problem (MTSP) are the two classical variants of the TSP, and they all generalize the TSP.

In the CTSP, all cities to be served are divided into several clusters. Compared to the TSP, this problem has an additional constraint that the cities within each cluster need to be served consecutively. It is easy to see that the CTSP degenerates to the TSP when the number of clusters is equal to 1 or each cluster only has one vertex.

In the MTSP, the number of salesmen increases from one to multiple. The problem requires computing a tour for each salesman such that all tours together can pass through all cities. If the weight of each tour is defined to be the sum of the lengths of all the edges in that tour, the objective of the problem is usually divided into two types, one is to minimize the sum of the weights of all tours (Min-Sum), and the other is to minimize the weight of the maximum weight tour (Min-Max). It is not hard to see that regardless of the type of the objective, if the number of salesmen is equal to 1, the MTSP also degenerates to the TSP.

Both the CTSP and the MTSP have a wide range of applications. For example, the CTSP can model relevant practical problems in the fields of vehicle routing [1], production manufacturing [2], computer program restructuring [3], cytological sample observation [4], integrated circuit testing [5], and so on. The MTSP can be used to solve related problems in the fields of printing press scheduling [6], load balancing [7], school bus routing [8], design of global navigation satellite system surveying networks [9], and so on.

For both the CTSP and the MTSP, there have been many studies using genetic algorithms to solve them. For example, Potvin and Guertin [10] presented a genetic algorithm combined with an edge recombination operator and a 2-opt search to solve the CTSP. Ding et al. [11] proposed a two-stage genetic algorithm for the CTSP. Ahmed [12] developed a hybrid genetic algorithm using sequential constructive crossover and a 2-opt search and a local search to the ordered CTSP in which the clusters are visited in the prespecified order. For the MTSP, Tang et al. [13] designed a genetic algorithm with a one-chromosome representation, Malmborg [14] and Park [15] presented a genetic algorithm with a two-chromosome representation, Carter and Ragsdale [16] proposed a genetic algorithm with a two-part chromosome representation, Brown et al. [17] and Singh and Baghel [18] proposed a grouping genetic algorithm-based approach, respectively.

In this paper, we consider the min-max clustered traveling salesmen problem (MMCTSP). Given cities divided into clusters and a certain number of traveling salesmen, the requirement of the MMCTSP is that, on the basis of the MTSP, each tour passes exactly through some clusters and the cities within each cluster need to be passed through consecutively. The problem aims to achieve a min-max type objective, which means to minimize the weight of the maximum weight tour.

It is easy to see that the MMCTSP is a multiperson variant of the CTSP and a vertices clustering variant of the MTSP as well. Therefore, the MMCTSP generalizes the CTSP and the MTSP, respectively. It can model more practical problems and has a wider range of practical applications. In the literature, the other two problems that are closely related to the MMCTSP are the min-max cycle cover problem (MMCCP) and the clustered vehicle-routing problem (CluVRP).

In the MMCTSP, if each cluster contains only one vertex or the vertex set is not divided into clusters, the corresponding problem is called the MMCCP in the literature. For this problem, when all tours have a common starting vertex, Frederickson et al. [19] proposed an approximation algorithm with an approximation ratio of (ρ+1−1/m), where ρ is the approximation ratio for solving the TSP and *m* is the number of traveling salesmen. For the MMCCP in which no starting vertex of any tour is specified, Xu et al. [20], Jorati [21], Yu and Liu [22] successively developed approximation algorithms with better approximation ratios.

The CluVRP is another problem that is closely related to the MMCTSP in the literature. The main differences between these two problems are as follows. Firstly, in the CluVRP, each customer (corresponding to the city) has a nonnegative demand, and each vehicle (corresponding to the traveling salesman) has the same capacity, and the sum of the demands of the customers visited by each tour in a feasible solution cannot exceed the vehicle capacity. Secondly, the objective of the CluVRP is the min–sum type, which means to minimize the sum of the weights of all tours. For the CluVRP, Battarra et al. [23] proposed two exact algorithms, branch and cut as well as branch and cut and price, and Vidal et al. [24], Exposito et al. [25], Defryn and Sörensen [26], Pop et al. [27], Hintsch and Irnich [28], etc. presented solution methods from the perspective of heuristic algorithms.

As can be seen from the above, the MMCTSP generalizes the MMCCP and is also a special case of the CluVRP if we neglect the objectives of the problems. In the MMCCP, if the cities or customers to be served are grouped into several clusters due to geographical location or priority constraint, then the MMCTSP arises. On the other hand, in the CluVRP, if the vehicle capacity is much greater than the customer demand and the objective focuses on customer satisfaction or the workload balance of vehicles, then the MMCTSP arises again.

As the MMCTSP generalizes the MMCCP and thus it generalizes the TSP, it is an NP-hard problem. For the NP-hard problem, there are three main types of solution methods in the literature; namely, an exact algorithm, an approximation algorithm and a heuristic algorithm. Recently, Bao et al. [29] considered two variants of the MMCTSP and designed approximation algorithms with constant approximation ratios, respectively. In this paper, a two-stage solution method based on a genetic algorithm is designed from the perspective of a heuristic algorithm according to the problem characteristics. Specifically, in the first stage, a TSP is abstracted for each cluster, and then a genetic algorithm for the TSP is applied to determine the visiting order of vertices within the cluster. In the second stage: Firstly, each cluster is considered as a node, and the distances between each two nodes is defined by combining the results of the first stage with a combination of greed and random, and an MTSP is then constructed. Finally, a grouping-based genetic algorithm for the MTSP has been applied to determine the assignment of clusters to salesmen and the visiting order of the assigned clusters for each salesman.

In the computational experiments, small-scale, medium-scale and large-scale instances were tested separately. The experimental results indicate that in the small-scale instances, compared with the exact results obtained by the CPLEX solver, the best results obtained by the proposed algorithm have a relative error of no more than 1.5%, but the solving time is significantly reduced; in the medium-scale and large-scale instances, our algorithm shows good solution performance compared with the two related two-stage solution strategies in the literature.

The remainder of the paper is organized as follows. In Section 2, we give a formal description and a hybrid integer programming formulation of the MMCTSP. The two-stage optimization method based on a genetic algorithm for solving the MMCTSP is described in Section 3 and the computational experiments and the achieved results are presented and discussed in Section 4. Finally, we draw our conclusions in Section 5.

## 2. Problem Description and Mathematical Modeling

**Figure 1:**
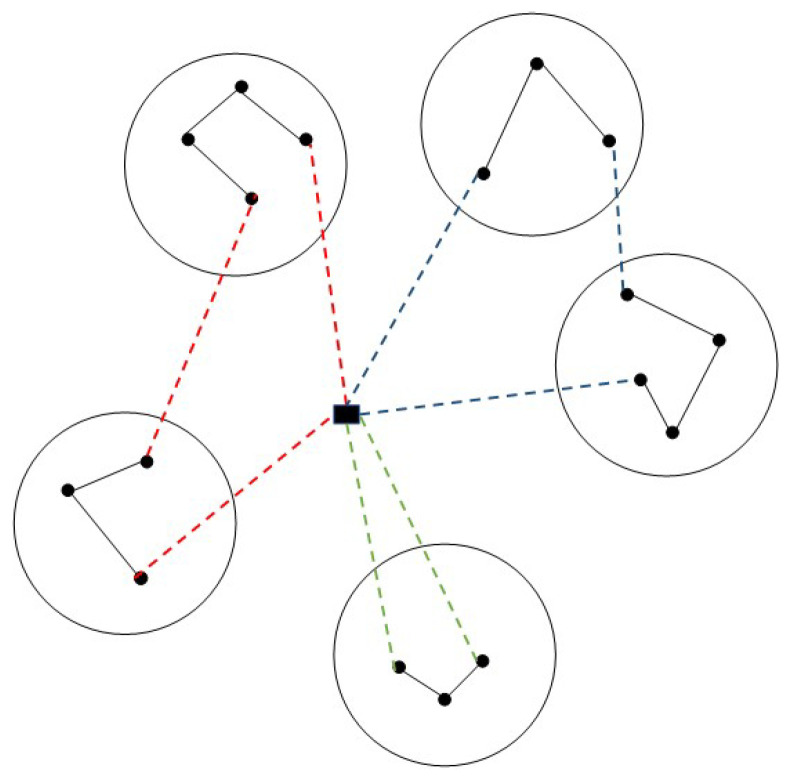
Schematic diagram of a feasible solution to the MMCTSP.

The MMCTSP can be described as follows: Given a complete undirected graph G=(V,E), V={1,2,⋯,n} is the vertex set, where 1 is the common starting vertex of all salesmen, and each vertex in the set {2,⋯,n} corresponds to a city. The vertex set *V* is partitioned into *l* clusters $V_{1},V_{2},\cdots ,V_{l}$, where $V_{1}=1$. Each edge (i,j) in the set *E* is associated with a non-negative real number $d_{ij}$ representing the distance between city *i* and city *j*. Given a set $E^{\prime }\subset E$, define the weight of $E^{\prime }$ as the sum of the lengths of all the edges in $E^{\prime }$. Given *m* salesmen, the MMCTSP requires computing a tour for each salesman such that all cities are visited and cities within each cluster are visited consecutively. The objective of the problem is to minimize the weight of the maximum weight tour. Figure 1 shows a schematic diagram of a feasible solution for the MMCTSP, where the dashed lines represent edges between clusters and between common starting vertex and clusters, while the solid lines indicate edges within clusters.

Next, the mathematical model of the MMCTSP is given first. The following decision variables are defined:

$$x_{ijk}=\left\{\begin{array}{cc} 1 & \mathrm{if}\;\mathrm{salesman}\;k\;\mathrm{travels}\;\mathrm{form}\;\mathrm{vertex}\;i\;\mathrm{to}\;\mathrm{vertex}\;j\\ 0 & \mathrm{otherwise} \end{array}\right.$$

$$y_{ik}=\left\{\begin{array}{cc} 1 & \mathrm{if}\;\mathrm{vertex}\;i\;\mathrm{is}\;\mathrm{visited}\;\mathrm{by}\;\mathrm{salesman}\;k\\ 0 & \mathrm{otherwise} \end{array}\right.$$

The hybrid integer linear programming model for this problem is:

objective function:

(1) $$min\;Z_{max}$$

subject to:

(2) $$\sum_{i=1}^{n}\sum_{j=1,j\neq i}^{n}d_{ij}x_{ijk}\leq Z_{max},k=1,2,\cdots ,m$$

(3) $$\sum_{k=1}^{m}\sum_{j=2}^{n}x_{1jk}=m$$

(4) $$\sum_{k=1}^{m}\sum_{j=2}^{n}x_{j1k}=m$$

(5) $$\sum_{k=1}^{m}y_{ik}=1,i=1,2,\cdots ,n$$

(6) $$\sum_{j=1,j\neq i}^{n}x_{ijk}=y_{ik},i=2,3,\cdots ,n,k=1,2,\cdots ,m$$

(7) $$\sum_{j=1,j\neq i}^{n}x_{jik}=y_{ik},i=2,3,\cdots ,n,k=1,2,\cdots ,m$$

(8) $$u_{i}-u_{j}+n\sum_{k=1}^{m}x_{ijk}\leq n-1,i,j=2,3,\cdots ,n,i\neq j$$

(9) $$\sum_{i,j\in V_{z},i\neq j}^{n}\sum_{k=1}^{m}x_{ijk}=\left|V_{z}\right|-1,z=1,2,\cdots ,l$$

(10) $$u_{i}\geq 0,i=2,3,\cdots ,n$$

(11) $$x_{ijk}\in \left\{0,1\right\},i,j=1,2,\cdots ,n;k=1,2,\cdots ,m$$

(12) $$y_{ik}\in \left\{0,1\right\},i=2,3,\cdots ,n;k=1,2,\cdots ,m$$

The objective function (1) represents the minimization of $Z_{max}$ which is given as an upper bound on travel distances of *m* salesmen in constraint (2). Constraints (3) and (4) ensure that each salesman starts and ends at vertex 1, constraint (5) requires each vertex to be visited by only one salesman, constraints (6) and (7) guarantee the continuity of route of each salesman, inequality (8) is the subtour elimination constraint, constraint (9) guarantees that vertices in each cluster are visited continuously, and constraints (10)–(12) represent constraints on the values of decision variables.

## 3. Algorithm Design

Due to the NP-hardness of the MMCTSP and the combinatorial complexity of the NP-hard problems, heuristic algorithms are an effective method of solving such problems for large-scale practical problems. Genetic algorithm is a common heuristic algorithm used in the literature for solving the TSP and the MTSP. For the MMCTSP, a two-stage solution method based on a genetic algorithm is proposed in this paper according to the problem characteristics and the rich literature results of applying genetic algorithms to solve problems closely related to it.

Given a feasible solution to the MMCTSP, each cluster is associated with a Hamiltonian path on that cluster, which gives the visiting order of the vertices within the cluster. To determine the visiting order of vertices within a cluster and enrich the connections between clusters, in the first stage of the proposed algorithm, we firstly abstract a TSP from each cluster. Then, we apply a genetic algorithm to solve the TSP and give the visiting order of vertices within a cluster (i.e., calculate a Hamiltonian cycle). To determine the assignment of clusters to salesmen and the visiting order of the assigned clusters, in the second stage of the proposed algorithm, by representing each cluster as a node and combining the results of the first stage and the ideas of greedy and random, we define the distances between each two nodes and construct an auxiliary MTSP, and then apply a grouping-based genetic algorithm to solve it.

### 3.1. Phase 1

**Figure 2:**
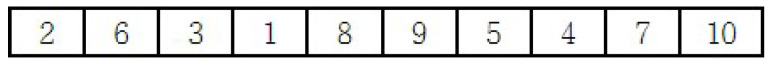
Chromosome encoding in the first stage.

For each cluster, the chromosome is encoded using natural numbers and the total number of genes in a chromosome is the number of all vertices within the cluster. In a chromosome, each gene represents a vertex and the gene order determines the order in which the vertices are visited. An example is given in Figure 2, where the tour is identified as:

2→6→3→1→8→9→5→4→7→10→2

In the genetic algorithm of this stage, the fitness function of each individual chromosome in the population is the inverse of the weight of the corresponding tour. For the genetic operators, we apply selection, crossover and mutation operators widely used in the literature. Specifically, we use the roulette wheel as selection operator, and apply the partial-matched crossover and the order crossover as crossover operators with equal probability, and adopt the swap mutation and the reverse mutation as mutation operators with equal probability. Meanwhile, we employ the elitist strategy during the population iteration.

### 3.2. Phase 2

Consider each cluster as a node of which the index corresponds to the cluster index. Next, we firstly provide the connection method between each two clusters, and then combine it with the result obtained in the first stage and present the distances between each two nodes, and finally construct an MTSP. By solving this MTSP, a feasible solution to the MMCTSP is obtained.

#### 3.2.1. Connections between Clusters

**Figure 3:**
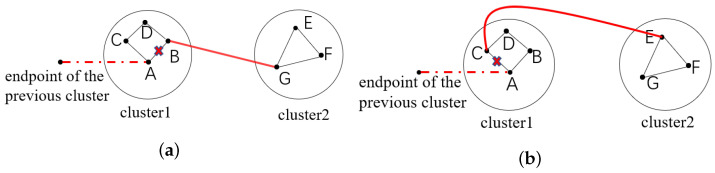
Schematic diagram of connections between clusters. (**a**) Strategy 1. (**b**) Strategy 2.

To enrich the feasible solutions obtained by our algorithm, two connection strategies between clusters are used with equal probability based on the TSP tour corresponding to each cluster. Given the initial vertex *A* of the previous cluster 1, the following explains how these two strategies are applied to determine the vertex leaving this cluster, using Figure 3 as an example.

The first strategy is to choose the vertex with the shortest distance to the subsequent cluster 2, among the two vertices *B* and *C* that are adjacent to *A*, as the vertex leaving this cluster. In Figure 3a, the distance from *B* to cluster 2 is the shortest, *B* is chosen as the vertex leaving cluster 1, and thus the visiting order of vertices within that cluster is A→C→D→B.

The second strategy is to randomly select vertex *B* or *C* as the vertex leaving this cluster. In Figure 3b, *C* is randomly selected as the vertex leaving cluster 1, so the visiting order of vertices within that cluster is A→B→D→C. Note that the vertex with the shortest distance from vertex *C* to the subsequent cluster 2 is *E*.

Next, we present the specific implementation process of applying a genetic algorithm to solve the MTSP. Since the objective function of the problem addressed in this paper is of min-max type, the grouping-based genetic algorithm exhibits better performance than a traditional genetic algorithm when solving this type of problem [17]. Inspired with the work of Singh and Baghel [18] and Han et al. [30], Wang et al. [31] proposed an improved grouping genetic algorithm and the associated genetic operators. Since the grouping genetic algorithm of Wang et al. [31] was originally designed to solve the min–sum type MTSP, we have made appropriate modifications to solve the min-max type MTSP in this stage.

#### 3.2.2. Chromosome Coding

**Figure 4:**
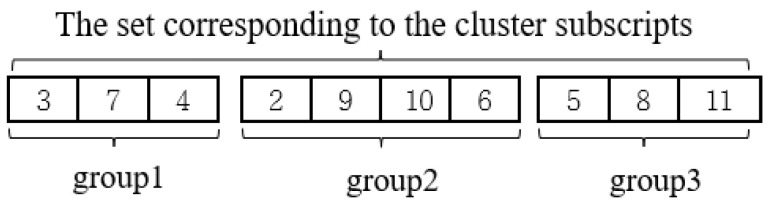
Chromosome encoding in the second stage.

In this stage, the chromosome is also encoded using natural numbers. The total number of genes in a chromosome is equal to the number of all clusters other than cluster $V_{1}$, and each gene represents a cluster. These genes are divided into *m* groups, and each group of genes determines the assignment of clusters to salesmen and the visiting order between clusters. The weight of the tour corresponding to each group is calculated, and the groups of genes are sorted in ascending order based on their weights. Figure 4 provides an example where salesman 1 visits clusters in the order of $V_{3}$, $V_{7}$ and $V_{4}$, salesman 2 visits clusters in the order of $V_{2}$, $V_{9}$, $V_{10}$ and $V_{6}$, and so on for salesman 3, and the weights of the corresponding tours increase from left to right.

#### 3.2.3. Fitness Function

The fitness function is the inverse of the weight of the maximum weight tour.

#### 3.2.4. Crossover Operator

The crossover operator is created through three steps, and each step is executed as follows.

**Figure 5:**
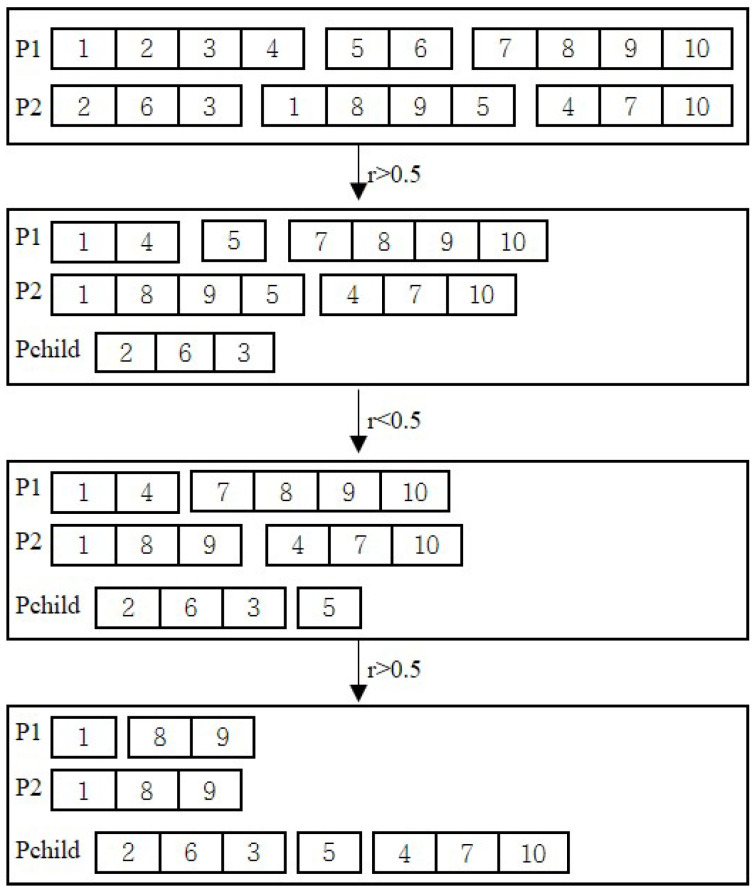
Schematic diagram of the first step of the crossover operator in the second stage.

In step 1, groups of genes are iteratively generated one by one from left to right until *m* groups of genes are constructed. When building the group of genes at position *i*, first, a random number r∈(0,1) is generated and if r<0.5, the group of genes at position *i* of the first parent is selected, otherwise the counterpart corresponding to the second parent is selected. Then the genes contained in that group of genes are removed from both parents, and the process continues to compute the next group of genes. Figure 5 provides an example of this process. Let n=10, m=3, and the two parents be denoted as P1 and P2, respectively.

P1={{1,2,3,4},{5,6},{7,8,9,10}},

P2={{2,6,3},{1,8,9,5},{4,7,10}}.

In step 2, in order to enhance the convergence accuracy of the algorithm, the greedy strategy and the 2-opt strategy are randomly applied to insert the unassigned genes into the offspring generated in step 1. The execution probabilities of the two strategies are $p_{1}$ and $1-p_{1}$, respectively.

In step 3, the weight value of the corresponding tour for each group of genes is first calculated and then all the groups of genes are sorted in ascending order according to their weight value to form a new offspring.

#### 3.2.5. Mutation Operator

Each gene from a parent is copied to the offspring with a probability $p_{2}$. For the unassigned genes, they are inserted into the previously calculated offspring by applying the same method as in step 2 of the crossover operator.

#### 3.2.6. Mutually Exclusive Execution

The crossover operator and the mutation operator are mutually exclusive with execution probabilities of $p_{3}$ and $1-p_{3}$, respectively.

### 3.3. Comparisons of Methods

In order to validate the effectiveness of the algorithm proposed in this paper, we compared it with the CPLEX solver and two related solution strategies in the literature, respectively. Specifically, the related comparisons were as follows.

Firstly, the results of the proposed algorithm were compared with those derived with the CPLEX solver for small-scale instances. For all eight test instances, the CPLEX solver provided exact solutions. Taking these results as a reference, the effectiveness of the proposed algorithm had been verified to some extent. The corresponding comparison is shown in Table 1.

For medium-scale and large-scale instances, due to the difficulty of obtaining better results in a shorter time using the CPLEX solver, as well as the lack of research results on the MMCTSP in the literature, two related solution strategies in the literature were compared with the algorithm proposed in this paper to verify its effectiveness.

The first solution strategy came from Exposito et al. [25]. This strategy was used to solve the CluVRP, referred to as the Clustered Capacitated VRP in [25], which is also a two-stage method. In the first stage, the concept of centroid was introduced to represent each cluster, a Capacitated VRP (CVRP) was generated and solved to determine the visiting order between clusters. In the second stage, for each cluster, based on the result obtained in the first stage, the visiting order of vertices within each cluster was determined, by solving a Hamiltonian path problem with two given endpoints, where the starting point was the endpoint of the route corresponding to the previous cluster and the endpoint was the centroid of the following cluster. To verify the good performance of the algorithm proposed in this paper in determining the visiting order between clusters and the visiting order of vertices within clusters, and to make the solution strategy in Exposito et al. [25] comparable to our algorithm, we generated the first comparison Algorithm A1 as follows. We first constructed an auxiliary MTSP based on the centroid informations, and then obtained the visiting order between clusters by applying the grouping-based genetic algorithm to solve it. Finally, we determined the visiting order of vertices within each cluster according to Exposito et al.’s algorithm.

The corresponding comparisons are shown in Table 2 and Table 3 for medium-scale and large-scale instances, respectively.

On the other hand, in order to verify the effectiveness of our algorithm in applying the grouping-based genetic algorithm for solving the MTSP constructed in the second stage, the second comparison Algorithm A2 was created as follows. We replaced the grouping-based genetic algorithm, in our algorithm, with the single-chromosome coding algorithm (with the same experimental parameters) proposed by Tang et al. [13] for solving the MTSP. Apart from this, there were no other changes. The corresponding comparisons are shown in Table 2 and Table 3 for medium-scale and large-scale instances, respectively.

## 4. Experimental Results and Analysis

### 4.1. Test Environment and Experimental Instances

This paper implemented the proposed algorithm using Matlab programming and ran it on a PC configured with a 64-core AMD Ryzen 7 4800H with Radeon Graphics @2.90 GHz (16 GB RAM). A total of 18 instances were selected from distance symmetric instances in TSPLIB in three sizes: small, medium and large. The instances were att48, st70, kroC100, rd100, ch130, ch150, kroB200, gr229, lin318, rd400, d493, att532, gr666, rat783, pr1002, d1291, fl1577 and d2103 with the number following each instance name representing the number of cities included in that instance. Computational experiments were conducted for each instance by dividing different clusters and setting different numbers of salesmen.

### 4.2. Parameter Determination

In the two-stage genetic algorithm designed in this paper, the parameters to be considered include: $p_{1}$, $p_{2}$ and $p_{3}$ in the second stage, population size, and the maximum number of iterations maxgen. The evaluation of the final solution obtained with the algorithm serves as a reference for parameter tuning. To experiment with parameter tuning, we fixed other parameters while adjusting one parameter, and conducted 10 experiments for each parameter. Finally, the parameters were determined as: $p_{1}=0.35$, $p_{2}=0.9$, $p_{3}=0.8$, size = 100, and maxgen = 500.

#### 4.2.1. Small-Scale Instances Experiments

In the small-scale instances experiments, for each size of instance, we used the same grouping method and generated a total of eight instances by setting the number of salesmen to 4 and 5, respectively. For the evaluation indicator, the best relative error $G_{Best}$ was used.

$$G_{Best}=\frac{I_{Best}-C_{Best}}{C_{Best}}.$$

Here, $C_{Best}$ represents the optimal value obtained with the CPLEX solver, and $I_{Best}$ denotes the best result obtained with the algorithm proposed in this paper for 10 times. The comparison between the results obtained with the proposed algorithm and those obtained with the CPLEX solver is shown in Table 1, where name represents the names of the instances, *n* is the number of cities, *m* is the number of traveling salesmen, *k* represents the number of clusters, and $I_{Average}$ denotes the average value obtained with the proposed algorithm for 10 times. Meanwhile, the bold numbers in the table represent the best results obtained with the CPLEX solver and the algorithm of this paper. From Table 1, it can be observed that:

1. In terms of computational accuracy, our algorithm achieved optimal results in $I_{Best}$ for six instances, including att48-4, att48-5, st70-4, st70-5, rd100-4 and rd100-5. Although $I_{Best}$ did not reach the optimal results in kroc100-4 and kroc100-5, the best relative error $G_{Best}$ was no more than 1.5%.
2. In terms of computational time, our algorithm exhibited significantly lower solving time than the CPLEX solver on all eight instances, indicating a clear advantage in computational efficiency.

Based on the above, our algorithm showed good solving performance on small-scale instances.

#### 4.2.2. Medium-Scale Instances Experiments: Comparisons with Algorithms A1 and A2

In the experiments of medium-scale instances, for each size of instance, the same grouping method was used, and the number of salesmen was set to 3, 5, and 10, respectively, resulting in 21 instances. The performance of our algorithm was compared with Algorithms A1 and A2 based on the experimental results presented in Table 2, where $I_{Best}$, $I_{Best}^{\prime }$, and $I_{Best}^{\prime \prime }$ represent the best results obtained by running our algorithm, Algorithms A1 and A2 10 times, respectively, and $I_{Average}$, $I_{Average}^{\prime }$, and $I_{Average}^{\prime \prime }$ represent the average results obtained by running our algorithm, Algorithms A1 and A2 10 times, respectively. Here, the bold numbers in the table represent the best results obtained with the algorithm of this paper, Algorithms A1 and A2. As can be seen from the table, in a total of 21 instances, our algorithm consistently outperformed Algorithms A1 and A2 in terms of computational accuracy for both the best and average results.

In Algorithm A1, the centroid of each cluster was computed first, then the visiting order between clusters was determined based on the information of all centroids, and finally the visiting order of vertices within each cluster was determined using the endpoint of the previous cluster and the centroid of the subsequent cluster. On the other hand, our algorithm determined the visiting order of vertices within each cluster first, and then used a combination of greedy and random ideas to determine the connecting edges between clusters and the visiting order between them based on this result.

In determining the visiting order between clusters, Algorithm A1 relied on the centroid information for each cluster, while our algorithm relied on the information of the visiting order of all vertices within each cluster. The latter allowed for a more comprehensive characterization of each cluster. Meanwhile, when considering the connecting edges between clusters, our algorithm employed both the greedy and random strategies, leading to a wider range of options for selecting connecting edges and increasing the diversity of solutions. Based on the above two factors, our algorithm exhibited a better solving performance.

For the MMCTSP, the solution method proposed in this paper was a two-stage strategy, where the second stage determined the visiting order between clusters by solving a constructed MTSP. For the MTSP, in comparison to Algorithm A2 based on a single chromosome encoding, our algorithm employed a grouping-based encoding scheme. The chromosomes generated with this encoding represented a feasible solution space with less redundancy, resulting in an improved search efficiency. Furthermore, the genetic operators and local search strategies under this encoding scheme further enhanced the convergence accuracy and search efficiency of the algorithm.

#### 4.2.3. Large-Scale Instances Experiments: Comparisons with Algorithms A1 and A2

The experimental results of our algorithm, Algorithms A1 and A2 for large-scales instances are shown in Table 3, where the bold numbers represent the best results obtained with the algorithm of this paper, Algorithms A1 and A2. It can be observed from the table that for a total of 21 instances in large scales, our algorithm outperformed Algorithms A1 and A2 in terms of computational accuracy for both the best and average results, demonstrating higher precision in the solutions.

## 5. Conclusions

In this paper, we considered the MMCTSP and proposed a two-stage solution method based on a genetic algorithm according to the characteristics of the problem. In the first stage, the visiting order of vertices within each cluster was determined by solving an abstracted TSP from the corresponding cluster. In the second stage, the visiting order between clusters was determined by solving an auxiliary MTSP. For both the TSP and the MTSP, we employed a genetic algorithm based methods to solve them, respectively. Computational experiments were conducted for instances of various scales. The experimental results demonstrated that our algorithm could obtain better solutions in a shorter time for small-scale instances, and exhibited a better computational performance compared to the two comparative algorithms for medium-scale and large-scale instances. In future research, it is worth paying attention to the corresponding routing problems where the service object is an edge or arc set of a given graph and all the service objects are divided into clusters.

## Figures and Tables

**Table 1:** Solution results for small-scale instances.

Instance Information				CPLEX		Algorithm of This Paper			Gaps
Name	*n*	*m*	*k*	$C_{\mathrm{Best}}$	Time (s)	$I_{\mathrm{Best}}$	$I_{\mathrm{Average}}$	Time (s)	
att48-4	48	4	8	**17,492**	8216	**17,492**	**17,492**	**5.21**	0.00
att48-5	48	5	8	**15,936**	4217	**15,936**	**15,936**	**5.21**	0.00
st70-4	70	4	10	**660**	6213	**660**	**660**	**7.28**	0.00
st70-5	70	5	10	**518**	3987	**518**	**518**	**7.30**	0.00
kroc100-4	100	4	10	**14,000**	13,762	14,210	14,482	**10.67**	0.015
kroc100-5	100	5	10	**10,836**	10,826	10,863	11,339	**10.69**	0.002
rd100-4	100	4	10	**4396**	24,676	**4396**	**4396**	**10.65**	0.00
rd100-5	100	5	10	**3643**	16,385	**3643**	**3643**	**10.74**	0.00

Note: Because a conventional performance notebook could not solve the above instances in a shorter time, in order to verify the effectiveness of the algorithm of this paper, $C_{Best}$ results were obtained by running the CPLEX program on a server configured with a 64-core Intel(R) Xeon(R) Gold 6130 CPU @ 2.10GHz (100GB RAM).

**Table 2:** Solution results for medium-scale instances.

Instance Information				Algorithm of This Paper			Algorithm A1			Algorithm A2		
Name	*n*	*m*	*k*	$I_{\mathrm{Best}}$	$I_{\mathrm{Average}}$	Time (s)	$I_{\mathrm{Best}}^{\prime }$	$I_{\mathrm{Average}}^{\prime }$	Time (s)	$I_{\mathrm{Best}}^{\prime \prime }$	$I_{\mathrm{Average}}^{\prime \prime }$	Time (s)
ch130-3	130	3	13	**2331**	**2425**	26.36	3076	3721	29.53	2764	2882	19.8
ch130-5	130	5	13	**1573**	**1639**	37.68	2176	2851	35.46	1983	2068	19.14
ch130-10	130	10	13	**1342**	**1404**	51.30	2239	2239	47.68	1492	1553	17.48
ch150-3	150	3	15	**6821**	**7212**	28.92	9184	10744	44.62	7669	7778	33.33
ch150-5	150	5	15	**4365**	**4451**	42.36	6224	7284	54.75	4874	4874	35.87
ch150-10	150	10	15	**3109**	**3158**	67.24	5518	6297	61.12	3733	3733	32.11
kroB200-3	200	3	20	**40,823**	**41,798**	36.21	56,364	65,108	52.82	42,806	44,451	42.75
kroB200-5	200	5	20	**25,062**	**25,831**	56.30	38,511	45,109	71.82	27,822	29,099	41.67
kroB200-10	200	10	2	**15,602**	**15,722**	92.77	22,329	28,783	79.14	17,423	18,347	40.73
gr229-3	229	3	23	**1654**	**1741**	41.15	3097	3509	59.41	1969	2027	35.63
gr229-5	229	5	23	**1061**	**1125**	61.42	2038	2507	73.63	1254	1316	34.84
gr229-10	229	10	23	**676.10**	**709.27**	103.17	1145	1531	78.06	722.29	796.53	38.60
lin318-3	318	3	32	**84,797**	**85,915**	57.64	104,810	133,910	87.39	88,476	89,740	73.48
lin318-5	318	5	32	**54,858**	**55,273**	81.56	72,122	81,894	87.30	56,093	58,220	70.35
lin318-10	318	10	32	**29,540**	**30,211**	159.41	41,242	51,173	112.46	31,866	34,450	66.00
rd400-3	400	3	40	**30,983**	**31,738**	68.98	39,959	43020	99.06	33253	33,483	85.53
rd400-5	400	5	40	**19,696**	**19,874**	100.82	25,287	28,449	124.17	20,663	21,480	83.78
rd400-10	400	10	40	**10,506**	**10,602**	194.65	14158	17,085	143.61	12,328	13,168	85.19
d493-3	493	3	49	**65,827**	**67,969**	115.84	93,084	113,684	129.72	71,669	72,592	108.73
d493-5	493	5	49	**44,124**	**44,714**	153.97	57,537	73,767	133.92	46,260	48,768	105.41
d493-10	493	10	49	**25,902**	**26,170**	236.36	35,458	41,956	169.01	29,055	30,506	105.72

**Table 3:** Solution results for large-scale instances.

Instance Information				Algorithm of This Paper			Algorithm A1			Algorithm A2		
Name	*n*	*m*	*k*	$I_{\mathrm{Best}}$	$I_{\mathrm{Average}}$	Time (s)	$I_{\mathrm{Best}}^{\prime }$	$I_{\mathrm{Average}}^{\prime }$	Time (s)	$I_{\mathrm{Best}}^{\prime \prime }$	$I_{\mathrm{Average}}^{\prime \prime }$	Time (s)
att532-3	532	3	53	**215,743**	**220,341**	119.71	289,158	318,530	138.06	228,991	232,097	116.04
att532-5	532	5	53	**137,012**	**138,704**	168.22	184,605	207,937	143.48	146,175	156,059	111.77
att532-10	532	10	53	**72,866**	**74,939**	263.21	98,817	124,253	181.24	80,924	91,853	109.59
gr666-3	666	3	67	**8065**	**8184**	154.44	10,995	11,756	175.88	8585	8633	142.70
gr666-5	666	5	67	**5116**	**5145**	203.38	6844	7863	170.93	5274	5668	140.14
gr666-10	666	10	67	**2713**	**2795**	322.30	4055	4688	238.86	3135	3378	140.87
rat783-3	783	3	78	**24,431**	**24,561**	184.07	30,672	33,129	185.00	25,240	25,668	172.77
rat783-5	783	5	78	**14,796**	**15,197**	237.54	19,637	21,021	239.58	15,551	16,721	170.97
rat783-10	783	10	78	**7817**	**7978**	374.08	11,152	12,747	266.16	9606	10,283	166.22
pr1002-3	1002	3	100	**984,119**	**994,034**	223.48	1,150,203	1,224,429	250.80	997,444	1,009,880	210.33
pr1002-5	1002	5	100	**596,696**	**608,471**	297.74	751,828	783,754	292.38	615,550	660,968	207.04
pr1002-10	1002	10	100	**312,674**	**315,691**	469.51	401,110	452,747	356.00	379,443	413,564	208.75
d1291-3	1291	3	129	**271,403**	**276,562**	295.72	324,725	34,473	344.27	278,225	282,548	283.36
d1291-5	1291	5	129	**168,879**	**170,007**	388.43	194,747	231,967	348.42	177,698	190,622	280.13
d1291-10	1291	10	129	**89,356**	**90,421**	606.09	116,666	157,610	472.67	98,753	114,074	287.97
fl1577-3	1577	3	158	**213,987**	**216,568**	357.20	256,092	271,006	414.86	216,275	218,102	350.05
fl1577-5	1577	5	158	**132,424**	**133,564**	474.69	158,467	184,102	420.36	137,305	143,273	341.93
fl1577-10	1577	10	158	**68,813**	**69,769**	754.05	117,114	125,894	581.34	83,239	90,810	345.49
d2103-3	2103	3	210	**503,707**	**507,482**	476.70	573,438	604,803	572.05	505,744	520,019	473.37
d2103-5	2103	5	210	**309,633**	**310,758**	632.80	348,375	379,010	572.48	318,063	343,450	463.39
d2103-10	2103	10	210	**159,885**	**161,239**	1007.44	186,445	266,479	763.19	190,118	212,572	454.45

## References

[1] Chisman J.A. (1975). The clustered traveling salesman problem. Comput. Oper. Res..

[2] Hoeft J., Palekar U.S. (1997). Heuristics for the plate-cutting traveling salesman problem. IIE. Trans..

[3] Horspool R.N., Laks J.M.S. (1983). An improved block sequencing method for program restructuring. J. Syst. Softw..

[4] Laporte G., Semet F., Dadeshidze V.V., Olsson L.J.S. (1998). A tiling and routing heuristic for the screening of cytological samples. J. Oper. Res. Soc..

[5] Laporte G., Palekar U. (2002). Some applications of the clustered travelling salesman problem. J. Oper. Res. Soc..

[6] Gorenstein S. (1970). Printing press scheduling for multi-edition periodicals. Manag. Sci..

[7] Okonjo-Adigwe C. (1988). An effective method of balancing the workload amongst salesmen. Omega.

[8] Angel R.D., Caudle W.L., Noonan R., Whinston A.N.D.A. (1972). Computer-assisted school bus scheduling. Manag. Sci..

[9] Saleh H.A., Chelouah R. (2004). The design of the global navigation satellite system surveying networks using genetic algorithms. Eng. Appl. Artif. Intell..

[10] Potvin J.Y., Guertin F. (1996). The clustered traveling salesman problem: A genetic approach. Meta-Heuristics: Theory and Applications.

[11] Ding C., Cheng Y., He M. (2007). Two-level genetic algorithm for clustered traveling salesman problem with application in large-scale TSPs. Tsinghua Sci. Technol..

[12] Ahmed Z.H. (2014). The ordered clustered travelling salesman problem: A hybrid genetic algorithm. Sci. World J..

[13] Tang L., Liu J., Rong A., Yang Z. (2000). A multiple traveling salesman problem model for hot rolling scheduling in Shanghai Baoshan Iron & Steel Complex. Eur. J. Oper. Res..

[14] Malmborg C. (1996). A genetic algorithm for service level based vehicle scheduling. Eur. J. Oper. Res..

[15] Park Y.B. (2001). A hybrid genetic algorithm for the vehicle scheduling problem with due times and time deadlines. Int. J. Prod. Econ..

[16] Carter A.E., Ragsdale C.T. (2006). A new approach to solving the multiple traveling salesperson problem using genetic algorithms. Eur. J. Oper. Res..

[17] Brown E.C., Ragsdale C.T., Carter A.E. (2007). A grouping genetic algorithm for the multiple traveling salesperson problem. Int. J. Inf. Tech. Decis..

[18] Singh A., Baghel A.S. (2009). A new grouping genetic algorithm approach to the multiple traveling salesperson problem. Soft. Comput..

[19] Frederickson G.N., Hecht M.S., Kim C.E. (1978). Approximation algorithms for some routing problems. Siam. J. Sci. Comput..

[20] Xu W., Liang W., Lin X. (2015). Approximation algorithms for min-max cycle cover problems. IEEE. Trans. Comput..

[21] Jorati A. (2013). Approximation Algorithms for Some Min-Max Vehicle Routing Problems. Master’s Thesis.

[22] Yu W., Liu Z. (2016). Improved approximation algorithms for some min-max and minimum cycle cover problems. Theor. Comput. Sci..

[23] Battarra M., Erdoğan G., Vigo D. (2014). Exact algorithms for the clustered vehicle routing problem. Oper. Res..

[24] Vidal T., Battarra M., Subramanian A., Erdoğan G. (2015). Hybrid metaheuristics for the clustered vehicle routing problem. Comput. Oper. Res..

[25] Expósito-Izquierdo C., Rossi A., Sevaux M. (2016). A two-level solution approach to solve the clustered capacitated vehicle routing problem. Comput. Ind. Eng..

[26] Defryn C., Sörensen K. (2017). A fast two-level variable neighborhood search for the clustered vehicle routing problem. Comput. Oper. Res..

[27] Pop P.C., Fuksz L., Marc A.H., Sabo C. (2018). A novel two-level optimization approach for clustered vehicle routing problem. Comput. Ind. Eng..

[28] Hintsch T., Irnich S. (2018). Large multiple neighborhood search for the clustered vehicle-routing problem. Eur. J. Oper. Res..

[29] Bao X., Xu L., Yu W., Song W. (2023). Approximation algorithms for the min-max clustered k-traveling salesmen problems. Theor. Comput. Sci..

[30] Han L., Wang Y., Lan S. (2010). Graph Coloring algorithm based on ordered partition encoding. Acta Electron. Sin..

[31] Wang Y., Chen Y., Yu Y. (2017). Improved grouping genetic algorithm for solving multiple traveling salesman problem. J. Electro. Inf. Tech..

